# Genes Controlled by DNA Methylation Are Involved in Wilms Tumor Progression

**DOI:** 10.3390/cells8080921

**Published:** 2019-08-17

**Authors:** João Victor da Silva Guerra, Bruna Maria de Sá Pereira, Jéssica Gonçalves Vieira da Cruz, Nicole de Miranda Scherer, Carolina Furtado, Rafaela Montalvão de Azevedo, Paulo Sergio Lopes de Oliveira, Paulo Faria, Mariana Boroni, Beatriz de Camargo, Mariana Maschietto

**Affiliations:** 1 Brazilian Biosciences National Laboratory (LNBio), Brazilian Center for Research in Energy and Materials (CNPEM), Campinas 13083-970, Brazil; 2 Graduate Program in Biosciences and Technology of Bioactive Products, Institute of Biology, University of Campinas, Campinas 13083-862, Brazil; 3 Brazilian National Cancer Institute (INCa), Rio de Janeiro 20231-050, Brazil; 4 Bioinformatics an Computacional Biology Lab, Brazilian National Cancer Institute (INCa), Rio de Janeiro 20231-050, Brazil; 5 Boldrini Children’s Hospital, Campinas 13083-884, Brazil

**Keywords:** Wilms tumor, cancer progression, DNA methylation, gene expression, data integration, DNMT, TET

## Abstract

To identify underlying mechanisms involved with metastasis formation in Wilms tumors (WTs), we performed comprehensive DNA methylation and gene expression analyses of matched normal kidney (NK), WT blastemal component, and metastatic tissues (MT) from patients treated under SIOP 2001 protocol. A linear Bayesian framework model identified 497 differentially methylated positions (DMPs) between groups that discriminated NK from WT, but MT samples were divided in two groups. Accordingly, methylation variance grouped NK and three MT samples tightly together and all WT with four MT samples that showed high variability. WT were hypomethylated compared to NK, and MT had a hypermethylated pattern compared to both groups. The methylation patterns were in agreement with methylases and demethylases expression. Methylation data pointed to the existence of two groups of metastases. While hierarchical clustering analysis based on the expression of all 2569 differentially expressed genes (DEGs) discriminated WT and MT from all NK samples, the hierarchical clustering based on the expression of 44 genes with a differentially methylated region (DMR) located in their promoter region revealed two groups: one containing all NKs and three MTs and one containing all WT and four MTs. Methylation changes might be controlling expression of genes associated with WT progression. The 44 genes are candidates to be further explored as a signature for metastasis formation in WT.

## 1. Introduction

Wilms tumor (WT) is an embryonic renal tumor with a median age adjusted incidence rate of 8.33 per million in Brazil [1]. The most frequently mutated genes in WT are *WT1* (12%), *AMER1* (18%), *CTNNB1* (15%), and *DROSHA* (12%) [2,3,4,5,6]. Genome-wide analysis in a large collection of tumors identified additional somatically mutated genes found less frequently, with most mutations often occurring in the same tumors. Currently, there are 37 genes found recurrently mutated in WT [7], which individually are not enough to cause tumor development in more than 5% of the patients. Although constitutional for 3% of the patients that do not present obvious clinical symptoms [8], around 70% of WT present imprinting abnormalities at the imprinting control region (ICR) of *IGF2/H19* [3] pointing to alterations in the mechanisms of gene expression regulation as being involved in WT formation [9]. WTs have lower LINE-1 methylation levels than paired adjacent normal kidney tissues, with tumors from patients that relapsed presenting even lower levels than those that did not relapse [10]. Methylation levels from CpG sites located close to genes or in intergenic regions divided WT in two subgroups, one with a methylation profile similar to nephrogenic rests and a second group presenting increased high methylation variability, hypomethylation of renal developmental genes, and hypermethylation of cell adhesion genes [11], this subgrouping still needs further confirmation.

Patients with WT are treated by either Children Oncology Group (COG) or International Society of Pediatric Oncology (SIOP) protocols, and had an overall survival rate of 90%, which strongly relies on the histological subtype and tumor stage [12]. The later include preoperative chemotherapy, followed by surgery and adjusted chemotherapy (radiotherapy for selected cases), according to tumor initial response [13,14]. The diverse WT histology stratifies patients in low (completely necrotic), intermediate (stromal, epithelial, mixed, and regressive tumors), and high (blastemal and diffuse anaplastic tumors) risks. While patients with low and intermediate risk tumors present 88% 5-year event free survival, patients with blastemal predominant tumors show 58% 5-year event free survival [15]. Diffuse anaplastic tumors have *TP53* abnormalities in 60% of the cases, present as an heterogeneous marker [16,17,18], which was associated with increased risk of recurrence and death [16]. There are several efforts in the search for biomarkers to improve the current risk stratification system [19,20,21]. Patients with WT might still be over- or under-treated and to predict those that will relapse remains difficult [22]. Avoiding the adverse treatment effects remains a challenge, especially considering the heterogeneous genetic susceptibility to other tumors, renal and heart diseases, among others [23,24,25].

Although DNA methylation and gene expression disruption were implicated in Wilms tumorigenesis, these mechanisms have not been explored in metastasis formation. Here, we explore DNA methylation together with gene expression in matched trios of normal kidney (NK), WT, and metastatic tissue (MT) to gain insight into the biology underlying WT progression.

## 2. Materials and Methods

### 2.1. Sample Collection and DNA and RNA Extraction

Patients were enrolled in the Brazilian National Institute of Cancer (INCa) into the International Society of Pediatric Oncology (SIOP) clinical trial 2001. All 110 cases (2003–2014) registered to the Department of Pathology were reviewed by a pathologist and a clinician that selected 11 cases (33 paired samples) that presented viable tissues in formalin-fixed paraffin-embedded (FFPE) blocks of matched normal kidney (cortex), WT (blastemal component), and metastatic tissues (all from lung). A total of 27 samples from nine cases passed quality control parameters for DNA and/or RNA integrity, five cases were evaluated by both methodologies, two cases were evaluated only for methylation experiments, and two cases only for expression experiments (Table 1). Microdissection was carried out by two punches of 1-mm core samples to maximize cellular homogeneity (>80% of blastemal cells). DNA and RNA from FFPE were extracted using QIAamp DNA Mini kit (Qiagen^®^, Hilden, Germany) and RecoverAll Total Nucleic Acid Isolation Kit for FFPE (Life Technologies, Carlsbad, CA, USA), respectively, following manufacture procedures. 

The Research Ethics Committee from INCA independently approved the study (ID 170/13), and informed consent was obtained from the patients’ legal guardians. All experiments were performed in compliance with the Helsinki guidelines.

### 2.2. Infinium HumanMethylation450 BeadChip Arrays (Illumina) Procedures

FFPE extracted DNA was recovered using Infinium HD FFPE Restore Protocol kit (Illumina, Inc, San Diego, CA, USA) and treated with EZ DNA Methylation Kit (Zymo Research Corp, Corporation, Irvine, CA, USA), following manufacturer’s guidelines. Thirty samples were profiled in the HumanMethylation450 BeadChip arrays (HM450K, Illumina), following the Illumina Infinium HD methylation protocol by Deoxi Biotecnologia (www.deoxi.com). Scanned HM450K (iScan SQ Scanner, Illumina) were processed into IDAT files by GenomeStudio software (v.2011.1, Illumina Inc, San Diego, CA, USA, 2011), with methylation module v.1.9.0 (Illumina). Probes were annotated according to the Illumina annotation file using the Human GRCh37/hg19. 

Quality control assessment pointed to two samples with low coverage and poor density profiles (Figure S1), resulting in the exclusion of two incomplete matched trios, leaving 21 matched samples (seven trios) for methylation experiments. Further steps implemented in minfi package [26] removed probes presenting unreliable fluorescence measurements, including multi-hit probes [27] (*n* = 6830), located in XYS (*n* = 52,017) and failed probes (detP > 0.05, *n* = 73,045). To best fit Infinium II to I probes, we applied Quantile [28] normalization on the remaining 353,620 probes (Figure S2). Methylation levels for each probe were shown as beta-values (0: unmethylated, 1: methylated), used for graphical representations; M-values (beta-values logit transformation) were used for statistical analysis due to the homoscedastic behavior, unless otherwise stated.

### 2.3. Methylation Statistical Analysis

To compare groups avoiding cellular heterogeneity effects, the sva package [29] was applied to estimate surrogate variables. Limma package [30] was used to generate an empirical Bayesian framework linear model [31] on matched trios, considering intra-patient and interpatient comparison. The estimated surrogate variables were considered covariables before identification of differentially methylated positions (DMPs). In the matched trio comparison, we considered significant those CpG sites with adjusted *p*-values < 0.001. In the pairwise comparisons, we considered significant those positions with an absolute mean beta change higher than 10% and *p*-value < 0.01. 

DMRcate was applied to identify differentially methylated regions (DMRs), defined as a 300 nucleotides sequence with at least seven CpG sites presenting methylation changes in the same direction [32]. Genomic regions with *p*-value < 0.05 and mean methylation differences greater than 10% (delta-beta > 0.1, hypermethylation) and smaller than 10% (delta-beta < −0.1, hypomethylation) were considered the top ones. DMRs were annotated for overlapping promoter regions, considering +/−2000 base pairs from transcription starting site of each gene. Functional annotation was performed by enrichment analysis using GREAT on the DMRs’ genomic regions [33].

### 2.4. RNA Library Construction and Sequencing

RNA samples were quantified by spectrophotometry using Nanodrop ND-2000 (Thermo Fisher Scientific, Waltham, MA, USA), followed by a quality control step by quantitative RT-PCR for *GAPDH* (114 nt). Sequencing libraries were generated with the TruSeq^®^RNA Access Library Prep (Illumina), appropriated for RNA extracted from FFPE tissues according to manufacturer’s procedures. We checked for sample quality at every step of analysis, discarding samples that did not reach the parameters. From 33 samples, 12 samples were excluded due to low quality, leaving 21 samples (7 matched trios) for RNA sequencing. For clustering, 2 nM libraries were denatured with 0.1M NaOH and diluted at 12 pM HT1 buffer (Illumina) and loaded in cBot. Libraries were sequenced in the HiSeq2500 (Illumina) and the quality of the fastq files was evaluated by FastQC v.0.11.5 (Babraham Institute, Babraham, Cambridge, UK) [34]. Low quality bases, (phred score < 33, minimum length = 36 pb), and adapter sequences were removed using Trimmomatic v0.36 (RWTH Aachen University, Aachen, Germany) [35], while poly-A tails were removed using Prinseq-lite v0.20.4 (University of Birmingham, Birmingham, UK) [36] followed by data quality re-evaluation by FastQC. Both paired and unpaired reads were processed to recover maximum reads sequenced. Unpaired reads refer to those that one of the sequenced reads were not used due to low quality according to the evaluated metrics, but we considered them as technical replicates. Then, reads were mapped to the GRCh38 reference genome (*Homo_sapiens*, Ensembl) using STAR v2.5.3a [37], after removing the pseudogenes from the reference genome annotation file, generating the alignment statistics (Tables S1 and S2). SAM files (Sequence Alignment/Mapping) were converted to BAM (Binary SAM) files using SAMtools [38] and visualized in the Integrative Genome Browser (IGV) 2.4.4 [39].

### 2.5. Gene Expression Analysis

We used the HTSeq v0.6.1 (University of Heidelberg, Heidelberg, Germany) [40] to identify the number of reads maping to each gene. Data were normalized with DESeq2 package (University of North Carolina, Chapel Hill, ND, USA) [41], which accounts for sequencing depth and generates an expression matrix containing all genes for the 21 matched samples. We considered as differentially expressed genes (DEG), those genes that presented False Discovery Rate (FDR) < 0.001 and lfcThreshold = 0.1.

## 3. Results

### 3.1. DNA Variability Suggests the Existence of Two Groups of Metastatic Tissues

**Figure 1:**
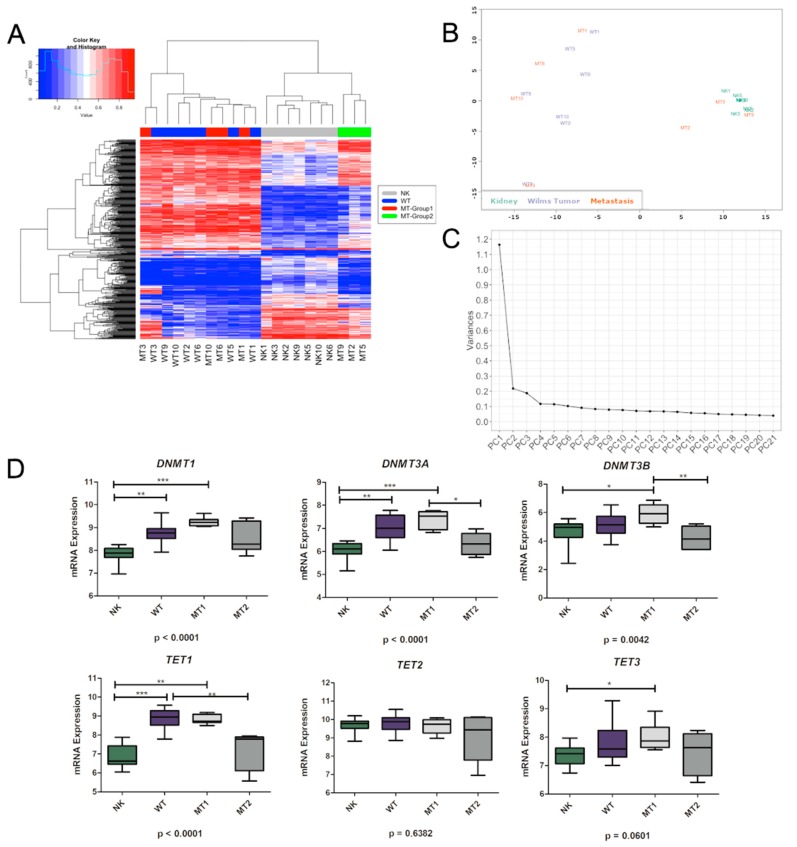
Methylation analyses in matched trios of normal kidney (NK), Wilms tumor (WT), and metastatic tissues (MT). (**A**) Hierarchical clustering (Euclidean distance with average linkage) of the 21 samples, based on methylation levels of the 497 differentially methylated CpG sites. Heatmap colors refer to methylation levels: unmethylated (blue), partially methylated (white), and methylated (red). (**B**) Multidimensional scaling of the top 1% most variable positions. (**C**) Variance in DNA methylation related to each principal component identified. (**D**) Boxplot representing expression levels (from RNAseq) for *DNMT1*, *DNMT3A*, *DNMT3B*, *TET1*, *TET2*, and *TET3*. Kruskal–Wallis test followed by Dunn post-test was applied: * *p* < 0.05, ** *p* < 0.01, *** *p* < 0.001. NK: normal kidney (*n* = 7), WT: Wilms tumor (*n* = 7), MT1: metastasis group 1 (*n* = 4), MT2: metastasis group 2 (*n* = 3).

DNA methylation for 353,620 CpG sites was evaluated in seven patients of matched NK, WT, and MT tissues. Comparison between matched trios (NK, WT, and MT) allowed the identification of 497 differentially methylated positions (DMPs; adjusted *p*-value < 0.001), with 345 DMPs located in 301 genes. Regarding to CpG island, 342 DMPs (69%) were located in CpG island, shores, or shelves whereas 155 DMPs (31%) were located in open sea (*p*-value < 0.0001; chi-squared test). Hierarchical clustering based on these DMPs (Euclidean distance with average linkage) resulted in two clusters: one containing all WT and four metastatic samples and a second containing all NK and three metastatic samples (Figure 1A). Likewise, methylation CpG levels show that NK, WT, and MT are distinct tissues and that there are two groups of metastases (MT-Group1: MT1, MT3, MT6, MT10, and MT-Group2: MT2, MT5, MT9). The level of similarity between samples using multidimensional scaling (MDS) applied to the 1% most variable sites (Figure 1B) showed that NK and three MT samples grouped tightly together (MT-Group2) and all WT and four MT samples (MT-Group1) showed high variability (*p*-value < 10^−16;^ Levene’s test). Consistently, we observed less variance at single CpG sites among NK (67.5%, *n* = 2386, s.d. < 0.1) than in WT (8.0%, *n* = 282, s.d. < 0.1) and within the metastatic samples, MT-Group2 (47.8%, *n* = 1692, s.d. < 0.1) showed less variance than MT-Group1 (22.7%, *n* = 804, s.d. < 0.1). To gain a comprehensive insight into the variation in DNA methylation between the three groups, we applied Principal Component Analysis (PCA) to the full dataset. The principal component (PC) 1 and 2 explained 88.1% and 3.1% of the variance (Figure 1C), respectively, which clearly separated NK and WT and both groups of metastases. This suggests that NK has a more stable epigenome than WT, consistent with previous reports in normal and cancer tissues. MT-Group1 had twice as many variant CpGs than MT-Group2, pointing to differences in the genomic stability between both groups. The histology of the metastatic samples as well as the clinicopathological characteristics of the tumors are presented in Table 1.

### 3.2. Methylation Differences May Be Related to DNMTs and TETs Expression

By applying pairwise comparisons between NK, WT, and MT groups, we identified 8860 hypo- and 8746 hypermethylated positions between WT and NK and 825 hypo- and 1314 hypermethylated DMPs between MT and WT (*p*-value < 0.01 and Delta beta-value (DB) > 10%). Most of the DMPs from WT and NK were located in CpGs island, shores, or shelves, independently of its methylation pattern (61% hypomethylated and 65% in hypermethylated DMPs), similar to the DMPs from MT and WT (59% hypomethylated and 66% in hypermethylated DMPs).

Further, we verified the expression of methylases and demethylases by looking for a correlation between methylation pattern observed in NK, WT, and MT, considering both MT-groups. *DNMT1*, *DNMT3A*, and *TET1* reported lower expression in NK compared to WT. MT-Group1 and MT-Group2 showed different expression levels for *DNMT3A* and *DNMT3B*, again with MT-Group1 showing expression levels similar to WT and MT-Group2 being more similar to NK (Figure 1D).

### 3.3. Genes Controlled by DNA Methylation Confirm the Existence of Two Groups of Metastases

**Figure 2:**
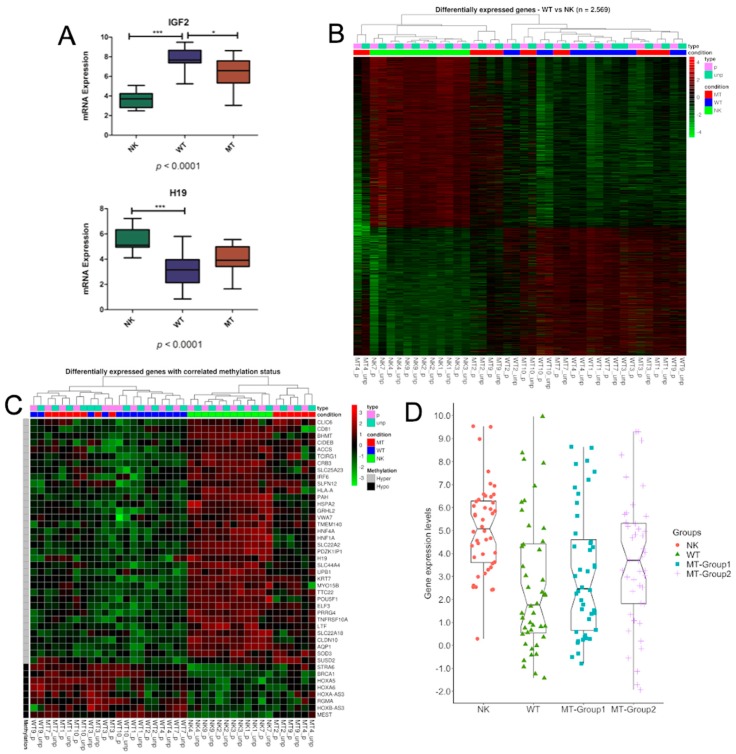
Genes controlled by DNA methylation. (**A**) Boxplot of expression levels across the groups (NK, WT, and MT) for *IGF2* and *H19* (ANOVA and Tukey’s Multiple Comparison Test; * *p* < 0.05, *** *p* < 0.001) (**B**) Hierarchical clustering (distance was measured as 1-Pearson correlation coefficient with complete linkage) of the paired seven cases (21 samples), based on expression levels of the (**B**) 2569 differentially expressed genes (DEGs) and (**C**) 44 genes controlled by methylation. Heatmap colors refer to expression levels Z-score transformed: lower expression (green), median levels partially methylated (black), and highly expressed (red). (**D**) Boxplot of expression levels across the groups (NK, WT, MT-Group1, and MT-Group2) for 44 genes controlled by methylation. NK: normal kidney (*n* = 7), WT: Wilms tumor (*n* = 7), MT: metastasis (*n* = 7), MT-Group1: metastasis group 1 (*n* = 4), MT-Group2: metastasis group 2 (*n* = 3).

To examine the relationship between methylation and RNA expression, we correlated the methylation levels of DMRs and the expression of the closest genes. By comparing WT and NK, we identified 92 hypo- and 207 hypermethylated regions in WT (*p*-value < 0.05 and DB > 10%), and 1.100 up and 1.469 downregulated genes (FDR < 0.001) in WT compared to NK, with 44 showing agreement between hypomethylation/over-expression (*n* = 8) and hypermethylation/down-expression (*n* = 36) (Table 2). Similar to previous studies [3,7], methylation of 11p15 ICR1 correlated with IGF2 and H19 expression (Figure 2A). Four genes from HOX family reported hypomethylation associated with over-expression: *HOXA5*, *HOXA6*, *HOXA-AS3*, and *HOXB-AS3*. Five genes are recognized tumor suppressor genes, the hypermethylation was associated to down-regulation for *LTF*, *SUSD2*, *HNF4A*, *TNFRSF10A*, and *H19*. The exception was BRCA1 that presented promoter hypomethylation but was over-expressed in WT. These findings suggest that DNA methylation could be a mechanism for loss of function in a subset of tumors. While hierarchical clustering analysis based on the expression of all 2569 differentially expressed genes (DEGs) discriminated WT and MT from all NK samples (Figure 2B), the hierarchical clustering based on the expression of 44 genes revealed two groups: one containing all NKs and three MTs and a second containing all WT and two MTs (Figure 2C).

Thus, we verified the expression of these 44 genes in the MT samples (Figure 2D; Figure S3). Overall, the 44 genes expression showed a higher variance in MT-Group1, similar to WT, and a smaller variance in MT-Group2, closer to NK, suggesting their involvement into different paths of tumor progression. 

### 3.4. Characterization of the DMRs within the Metastatic Groups

To investigate how the two MT groups differentiate, we then established which methylated regions primarily discriminated between both MT groups and their matched WT. We identified 13 hypo- and 21 hypermethylated regions between MT-Group1 and WT, located in 11 (13 genes) and 19 (26 genes) promoters. These DMRs were enriched for five molecular functions: antigen binding, *TAP1* and *TAP2* binding, peptide antigen binding and peptide-transporting ATPase activity (FDR < 0.01; Table S3) and no biological processes. DMRs with the higher methylation differences (>20%) were located at the promoter regions of *RUSC1* (and *RUSC1-AS1*), *KCNQ1DN*, both more methylated in MT-Group1, *DNHD1* and *EXOC3L2*, both more methylated in WT.

The comparison between MT-Group2 and WT reported 130 hypo- and 36 hypermethylated regions, located in 118 (155 genes) and 30 (38 genes) promoters. These DMRs were enriched for two molecular functions: peptide antigen binding and MHC class II receptor activity (Table S4); and for several biological processes including antigen processing and presentation of exogenous antigen, genetic imprinting, interferon-gamma-mediated signaling pathway, meiosis, and regulation of T cell mediated cytotoxicity (FDR < 0.01, Table S5). All the DMRs with the higher methylation differences (>40%) were more methylated in WT than in MT-Group2, with one not located in gene promoter and 10 located the promoter of *HIST1H4I*, *HCG9*, *SALL4*, *CACNA1C-AS1*, *GRIK2*, *SIM2*, *ELTD1*, *ZNF300P1*, *NKAPL*, *ZKSCAN4*, and *RP11-573G6*.

To verify again the similarities between MT-Group2 and NK, we compared the expression levels between both MT groups identifying 2880 and 598 DEGs between MT-Group1 and MT-Group2, respectively, versus NK. This analysis showed that MT-Group1 has 5.8 more DEGs with 425 (71%) common genes between both comparisons.

## 4. Discussion

We investigated global gene expression and DNA methylation in case-matched triplets (normal tissue, primary cancer, and lung metastasis) to apply a comprehensive analysis of WT disease progression. Both variance and differential methylation analyses pointed to the existence of two groups of metastases. The methyltransferases *DNMT3A* and *DNMT3B* also reported differential expression between both groups. Similar to findings in other cancers [42], tumor samples presented high variance whereas normal samples clustered together, with the metastatic tissues showing low or high variance, similar to normal or tumor tissues. These groups were not identified in the expression data when all DEGs were considered. Nevertheless, using only DEGs that had a DMR located in the respective promoter, the clustering analysis identified the two groups of metastatic samples. We selected areas containing blastema (>80% of the cells); however, we cannot exclude the possibility of contamination by other cell types, resulting in, at least partially, differences in DNA methylation and gene expression.

MT-Group1 and MT-Group2 exhibited distinct methylation and expression patterns with MT-Group2 being more similar to NK suggesting that alterations in DNA methylation rather than acquisition of mutations are involved with metastasis. This difference was also observed at expression levels that showed that MT-Group1 had almost six more differentially expressed genes than MT-Group2, compared to NK. Epigenetic patterns were described within tumor types and associated to distinct clinical and pathological characteristics, such as age to diagnosis, sex, and relapse [42,43], what could related to the existence of both metastatic groups, however, a larger cohort is necessary to characterize the subgroups. Another hypothesis is that MT-Group1, that preserves WT alterations is not yet completely adapted to the new microenvironment while samples from MT-Group2 are already established in the lung presenting methylation and expression levels related to the survival in a fully differentiated and functional organ.

Changes during tumor progression were analyzed using DMRs from matched primary and normal tissues, which were then individually evaluated in metastatic samples. We found that a minority (14.7%, 44 out of 299) of the DMRs showed negative correlation between methylation and expression levels. This relatively low correspondence may be explained by the fact that we only used DMRs located in gene promoters. A downstream (up to 8 kilobases (kb)) or upstream (−2 kb to 0.3 kb) region away from the promoter seems to have a stronger correlation [44]. Nevertheless, we found two out three DMRs previously found hypermethylated in WT compared to NK: chr6:28956259-28956804 and chr6:32115979-32117565 located in *HCG16* and *PRRT1*, respectively [45]. Control of *H19* and *IGF2* expression by methylation and their association with WT is well described [3,46,47,48], thus validating our other findings. Most of the 44 genes were not previously described in WT but they have been associated to tumor progression and resistance to chemotherapy treatment in other cancers. Therefore, they might be of clinical interest for stratification of patients into high and low metastatic risk as well as to disclose potential targets for development of new therapies.

It is likely that epigenetic regulation of gene expression plays an important role in regulating the expression of transcription factors that determine progenitor self-renewal and/or nephron differentiation. PRC2 is recruited to unmethylated CpG sites to place the H3K27me3 mark at promoter and enhancer regions, resulting in transcriptional repression and nucleosome compaction. Repression of PRC2 (and PRC1) is believed to be a major mechanism whereby gene expression is negatively regulated during development, including HOX and SOX genes [49]. The HOX family are well-known genes with roles in development, differentiation, and motility, with their aberrant expression related to epigenetic alterations in tumorigenesis. In normal differentiated tissues, HOX genes are usually methylated, which is lost in tumors, together with the increase in expression, as demonstrated in myeloid/lymphoid or mixed lineage leukemia [50]. In our study, *HOXA5*, *HOXA6, HOXA-AS3*—all in the same cluster—and *HOXB-AS3* showed negative correlation between expression and methylation.

Some tumor suppressor genes may have lost their function in WT by disruption of DNA methylation in their promoter regions. *HNF4A* was involved with regulation of embryonic development of multiple tissues, including renal development [51]. Little is known about the epigenetic mechanism of this gene regulation, but in young-adult mouse liver *HNF4A* deficiency alters histone methylation and acetylation [52]. In humans, changes in *HNF4A* expression were associated with liver, colon, and hepatocellular carcinoma tumorigenesis [53]. *TNFRSF10A* is a receptor activated by tumor necrosis factor-related apoptosis inducing ligand *TNFSF10*, signaling for cell apoptosis. Its suppression is associated with inactivation of apoptotic pathways and consequently to tumor development in osteosarcomas [54], gastric carcinomas [55], and glioblastoma multiforme [56,57]. *SUSD2* is type I membrane protein containing domains inherent to adhesion molecules, in which downregulation was associated with proliferative capacity renal cell and lung carcinomas [58] and other cancers [59,60]. *SUSD2* interacts with *GAL1* to promote tumor immune evasion, angiogenesis, and metastasis [59]. *LTF* also has an immune regulatory function. The association of genes regulated by DNA methylation with tumor progression may reveal new possibilities to investigate new mechanisms and treatment possibilities for WT. 

Here, we explored modification of DNA methylation with respect to normal tissues and associated with cis-changes of gene expression. While functional investigations of specific targets will be required to validate cancer specificity and causal relationships of epigenetic and transcriptional changes, DNA methylation signatures could be used as a tool to evaluate as tumor progression, similar to prostate cancer [61]. In addition to the *IGF2/H19* cluster, possible candidates include those genes from the HOX family and the tumor suppressors. Overall, because DNA methylation constitutes a mechanism of gene expression control, systematic investigations on how cancer cells exploit this mechanism to deregulate specific targets and processes can help us to understand the disease manifestation, by capturing and functionally implicating cancer-associated methylation events and exploiting the therapeutic opportunities [62]. In WT, the response of the lung nodules to chemotherapy is used to modify treatment intensity, with the use of an additional agent, Irinotecan, being explored as a new strategy for metastatic and relapsed WT [63].

## 5. Conclusions

The differences in DNA methylation reported for NK, WT, and MT may be a result of the differential expression of methylases and demethylases. Methylation data pointed to the existence of two groups of metastases. These methylation changes may be controlling the expression of genes related to the metastasis formation in WT. In particular, the 44 genes are candidates to be further explored as a signature for metastasis formation in WT.

## Figures and Tables

**Table 1:** Clinicopathological characteristics of the cases evaluated for DNA methylation and gene expression.

Sample (ID)	Age at Diagnosis (years)	Gender	Histology (Primary Tumor)	Patient Stage	Relapse Site	Histology (Metastasis)	Technique
1	3	F	Blastemal	III	Left lung	Mixed	RNA-Seq/450k
2	7	M	Regressive	II	Left lung	Epithelial	RNA-Seq/450k
3	5	M	Mixed	I	Left lung	Blastemal	RNA-Seq/450k
4	3	F	Mixed	II	Right lung	Blastemal	RNA-Seq
5	3	F	Mixed	II	Right and left lung	Epithelial	450k
6	9	M	Mixed	I	Left lung	Mixed	450k
7	4	M	Regressive	I	Right lung	Blastemal	RNA-Seq
9	3	M	Mixed	I	Right lung	Blastemal	RNA-Seq/450k
10	6	M	Mixed	I	Right lung	Mixed	RNA-Seq/450k

Gender: female (F) and male (M).

**Table 2:** List of 44 genes showing negative correlation between promoter methylation and gene expression levels.

Gene	DMR Location	Number of CpGs	DMR Width (Bp)	Minimum *p*-value	Methylation Status (MaxBetaFC)	Methylation Status (MeanBetaFC)	Expression (Log2FC)
*ACCS*	chr11:44087396-44088257	12	862	5 × 10^−19^	0.5	0.3	−1.4
*AQP1*	chr7:30951064-30951801	11	738	6 × 10^−18^	0.4	0.1	−3.9
*BHMT*	chr5:78407153-78407683	8	531	4 × 10^−40^	0.5	0.4	−7.5
*BRCA1*	chr17:41277974-41279022	21	1049	2 × 10^−25^	−0.5	−0.2	2.2
*CD81*	chr11:2397255-2398336	21	1082	2 × 10^−46^	0.4	0.2	−1.2
*CIDEB*	chr14:24779793-24780926	13	1134	4 × 10^−43^	0.6	0.2	−2.2
*CLDN10*	chr13:96204518-96204978	8	461	1 × 10^−4^	0.2	0.1	−5.6
*CLIC6*	chr21:36041334-36041699	8	366	1 × 10^−52^	0.6	0.5	−3.0
*CRB3*	chr19:6463949-6464275	9	327	1 × 10^−23^	0.4	0.3	−5.3
*ELF3*	chr1:201979478-201979938	7	461	1 × 10^−6^	0.3	0.1	−4.8
*GRHL2*	chr8:102504447-102504859	8	413	7 × 10^−16^	0.3	0.2	−4.0
*H19*	chr11:2019452-2020560	29	1109	4 × 10^−12^	0.3	0.1	−2.1
*HLA-A*	chr6:29910411-29911095	8	685	4 × 10^−18^	0.4	0.2	−2.2
*HNF1A*	chr12:121416315-121416796	7	482	7 × 10^−10^	0.4	0.2	−4.9
*HNF4A*	chr20:42983920-42984878	12	959	1 × 10^−17^	0.4	0.2	−5.4
*HOXA-AS3*	chr7:27183816-27185512	26	1697	5 × 10^−16^	−0.3	−0.2	2.6
*HOXA5*	chr7:27183816-27185512	26	1697	5 × 10^−16^	−0.3	−0.2	1.8
*HOXA6*	chr7:27183816-27185512	26	1697	5 × 10^−16^	−0.3	−0.2	2.3
*HOXB* *-AS3*	chr17:46669455-46670029	9	575	4 × 10^−16^	−0.3	−0.1	2.7
*HSPA2*	chr14:65006688-65007437	16	750	1 × 10^−15^	0.4	0.2	−3.9
*IRF6*	chr1:209979111-209979779	9	669	3 × 10^−22^	0.5	0.3	−2.8
*KRT7*	chr12:52626814-52627576	8	763	1 × 10^−8^	0.4	0.2	−5.8
*LTF*	chr3:46506104-46506554	9	451	3 × 10^−5^	0.3	0.2	−7.0
*MEST*	chr7:130130753-130131730	13	978	3 × 10^−9^	−0.2	−0.1	3.7
*MYO15B*	chr17:73583839-73584360	9	522	1 × 10^−7^	0.3	0.2	−1.6
*PAH*	chr12:103310839-103311761	9	923	8 × 10^−20^	0.3	0.2	−8.1
*PDZK1IP1*	chr1:47655599-47656423	7	825	7 × 10^−7^	0.3	0.2	−7.3
*POU5F1*	chr6:31148404-31148748	7	345	1 × 10^−6^	0.2	0.1	−4.7
*PRRG4*	chr11:32851087-32851531	9	445	1 × 10^−4^	0.2	0.1	−4.2
*RGMA*	chr15:93616894-93617168	11	275	2 × 10^−9^	−0.3	−0.2	2.7
*SLC22A18*	chr11:2925594-2925969	8	376	2 × 10^−4^	0.2	0.1	−2.5
*SLC22A2*	chr6:160679391-160680162	10	772	3 × 10^−13^	0.3	0.2	−8.0
*SLC25A23*	chr19:6463949-6464275	9	327	1 × 10^−23^	0.4	0.3	−1.6
*SLC44A4*	chr6:31846769-31847028	8	260	1 × 10^−7^	0.2	0.2	−4.1
*SLFN12*	chr17:33759512-33760527	11	1016	1 × 10^−14^	0.5	0.3	−1.7
*SOD3*	chr4:24796689-24797176	7	488	1 × 10^−3^	0.2	0.1	−2.3
*STRA6*	chr15:74494781-74495354	7	574	4 × 10^−15^	−0.3	−0.2	3.6
*SUSD2*	chr22:24577223-24577448	7	226	4 × 10^−3^	0.3	0.1	−2.7
*TCIRG1*	chr11:67806118-67806668	7	551	3 × 10^−30^	0.6	0.4	−2.2
*TMEM140*	chr7:134832544-134833299	7	756	9 × 10^−10^	0.4	0.2	−3.1
*TNFRSF10A*	chr8:23082634-23082961	7	328	2 × 10^−4^	0.3	0.2	−3.1
*TTC22*	chr1:55266296-55267152	8	857	1 × 10^−7^	0.3	0.2	−5.7
*UPB1*	chr22:24891141-24891666	8	526	2 × 10^−10^	0.3	0.2	−3.1
*VWA7*	chr6:31740805-31741184	8	380	1 × 10^−9^	0.2	0.2	−1.7

## Supplementary Materials

The following are available online at https://www.mdpi.com/2073-4409/8/8/921/s1, Table S1: Alignment statistics to the reference genome mapping obtained by the STAR program, for paired reads, Table S2: Alignment statistics to the reference genome mapping obtained by the STAR program, for unpaired reads, Table S3: Molecular functions enriched in differentially methylated regions between MT-Group1 and WT by the GREAT software, Table S4: Molecular functions enriched in differentially methylated regions between MT-Group2 and WT by the GREAT software, Table S5: Biological processes enriched in differentially methylated regions between MT-Group2 and WT by the GREAT software, Figure S1: Quality control for Illumina Infinium Human Methylation 450k arrays. (A) Mean detection *p*-values for each sample. (B) Density ‘bean’ plots (beta-values) for each sample, Figure S2: Density plots (beta-values) for raw data (left panel) and Quantile normalization procedure (right panel), Figure S3: Boxplots of gene expression levels of NK, WT, MT-Group1, and MT-Group2 per gene with negative correlated between methylation and expression.
