# Supplementary material for: Genes Controlled by DNA Methylation Are Involved in Wilms Tumor Progression
Source: Cells. 2019 Aug 17;8(8):921. doi: 10.3390/cells8080921 (PMC6721649; doi:10.3390/cells8080921)

Genes Controlled by DNA Methylation Are Involved in Wilms Tumor Progression

João Victor da Silva Guerra [1,2,†], Bruna Maria de Sá Pereira [3,†], Jéssica Gonçalves Vieira da Cruz [4], Nicole de Miranda Scherer [4], Carolina Furtado [3], Rafaela Montalvão de Azevedo [3], Paulo Sergio Lopes de Oliveira [1], Paulo Faria [3], Mariana Boroni [4], Beatriz de Camargo [3] and Mariana Maschietto [1,5,*]

[1] Brazilian Biosciences National Laboratory (LNBio), Brazilian Center for Research in Energy and Materials (CNPEM), Campinas 13083-970, SP, Brazil.

[2] Graduate Program in Biosciences and Technology of Bioactive Products, Institute of Biology, University of Campinas, Campinas 13083-862, SP, Brazil

[3] Brazilian National Cancer Institute (INCa), Rio de Janeiro 20231-050, RJ, Brazil

[4] Bioinformatics an Computacional Biology Lab, Brazilian National Cancer Institute (INCa), Rio de Janeiro 20231-050, RJ, Brazil

[5] Boldrini Children's Hospital, Campinas 13083-884, SP, Brazil (current institution)

* Correspondence: marianamasc@gmail.com; Tel.: +55-19-3787-9087 (M.M.)

† These authors contributed equally to this work.

Table S1. Alignment statistics to the reference genome mapping obtained by the STAR program, for paired reads.

Sample (ID)	Number of initial sequences	Unique Reads mapped (%)	Average Reads lenght	Short reads unmapped	Other unmapped reads
**WT1**	28.025.155	24.182.593 (86.29%)	211	2.74%	0.96%
**MT1**	9.591.150	7.758.792 (80.90%)	207	2.78%	1.92%
**NK1**	8.265.947	7.055.097 (85.35%)	208	3.81%	0.42%
**WT2**	15.218.151	13.301.437 (87.41%)	209	3.28%	0.55%
**MT2**	16.045.791	14.065.952 (87.66%)	211	1.68%	0.77%
**NK2**	14.397.634	12.238.664 (85.00%)	209	3.55%	0.56%
**NK4**	12.434.782	10.712.886 (86.15%)	207	2.15%	0.56%
**WT4**	23.660.671	20.342.233 (85.97%)	210	2.50%	0.77%
**MT4**	185.114	52.589 (28.41%)	210	61.51%	0.71%
**WT3**	8.442.282	6.977.104 (82.64%)	210	5.04%	0.53%
**NK3**	11.460.235	9.861.924 (86.05%)	210	2.97%	0.47%
**MT3**	27.712.503	24.085.823 (86.91%)	210	2.19%	0.44%
**MT9**	18.042.218	14.639.757 (81.14%)	210	3.48%	1.15%
**WT9**	10.415.604	8.002.971 (76.84%)	207	4.47%	1.41%
**NK9**	23.261.931	19.782.725 (85.04%)	209	2.12%	0.58%
**WT10**	25.643.281	21.089.735 (82.24%)	205	3.82%	1.12%
**NK10**	1.298.391	646.954 (49.83%)	194	35.12%	1.50%
**MT10**	5.248.212	3.604.552 (68.68%)	201	9.35%	2.58%
**MT7**	27.367.454	22.252.482 (81.31%)	207	5.66%	1.20%
**NK7**	2.443.739	1.983.430 (81.16%)	208	8.13%	0.42%
**WT7**	30.521.015	25.892.161 (84.83%)	208	4.71%	0.55%

Table S2. Alignment statistics to the reference genome mapping obtained by the STAR program, for unpaired reads.

Sample (ID)	Number of initial sequences	Unique Reads mapped (%)	Average Reads lenght	Short reads unmapped	Other unmapped reads
**WT1**	2.748.744	2.214.144 (80.55%)	88	3.67%	1.28%
**MT1**	967.847	709.517 (73.31%)	86	4.27%	2.21%
**NK1**	924.950	740.946 (80.11%)	87	3.94%	0.63%
**WT2**	1.495.001	1.220.704 (81.65%)	87	3.31%	0.66%
**MT2**	1.447.667	1.186.405 (81.95%)	87	3.32%	0.95%
**NK2**	1.622.292	1.298.085 (80.02%)	88	3.64%	0.82%
**NK4**	1.175.420	946.412 (80.52%)	86	4.46%	0.72%
**WT4**	2.439.502	1.963.812 (80.50%)	87	3.06%	0.93%
**MT4**	22.652	5.195 (22.93%)	89	67.27%	0.66%
**WT3**	761.800	516.179 (67.76%)	87	8.63%	1.47%
**NK3**	1.067.482	856.091 (80.20%)	88	5.80%	0.58%
**MT3**	2.401.760	1.838.745 (76.56%)	87	4.89%	1.03%
**MT9**	1.501.047	1.128.812 (75.20%)	89	5.63%	1.48%
**WT9**	1.010.016	640.502 (63.42%)	86	6.67%	2.38%
**NK9**	2.178.095	1.726.758 (79.28%)	88	3.81%	0.81%
**WT10**	3.757.967	2.514.690 (66.92%)	87	3.50%	2.30%
**NK10**	309.829	116.111 (37.48%)	84	25.79%	2.64%
**MT10**	749.482	449.503 (59.98%)	88	17.46%	2.01%
**MT7**	2.448.149	1.890.379 (77.22%)	89	5.25%	1.08%
**NK7**	256.934	190.548 (74.16%)	90	12.21%	0.65%
**WT7**	3.187.986	2.423.621 (76.02%)	87	3.36%	1.36%

Table S3. Molecular functions enriched in differentially methylated regions between MT-Group1 and WT by the GREAT software.

Term name	Binom FDR Q-value	Binom Fold Enrichment	Binom Observed Region Hits	Binom Region Set Coverage	Hyper FDR Q-value	Hyper Fold Enrichment	Hyper Observed Gene Hits	Hyper Total Genes	Binom Gene Set Coverage
**antigen binding**	5×10^{-3}	51	4	12%	7×10^{-4}	34	5	56	10%
**TAP2 binding**	4×10^{-3}	960	2	6%	5×10^{-2}	188	2	4	4%
**TAP1 binding**	4×10^{-3}	960	2	6%	5×10^{-2}	188	2	4	4%
**peptide antigen binding**	4×10^{-3}	97	3	9%	1×10^{-3}	68	4	22	8%
**peptide-transporting ATPase activity**	4×10^{-3}	594	2	6%	4×10^{-2}	150	2	5	4%

Table S4. Molecular functions enriched in differentially methylated regions between MT-Group2 and WT by the GREAT software.

Term name	Binom FDR Q-value	Binom Fold Enrichment	Binom Observed Region Hits	Binom Region Set Coverage	Hyper FDR Q-value	Hyper Fold Enrichment	Hyper Observed Gene Hits	Hyper Total Genes	Binom Gene Set Coverage
**peptide antigen binding**	1×10^{-6}	46	7	4%	8×10^{-3}	17	5	22	2%
**MHC class II receptor activity**	3×10^{-4}	77	4	2%	8×10^{-3}	25	4	12	2%

Table S5. Biological processes enriched in differentially methylated regions between MT-Group2 and WT by the GREAT software.

Term name	Binom FDR Q-value	Binom Fold Enrichment	Binom Observed Region Hits	Binom Region Set Coverage	Hyper FDR Q-value	Hyper Fold Enrichment	Hyper Observed Gene Hits	Hyper Total Genes	Hyper Gene Set Coverage
antigen processing and presentation of exogenous antigen	4×10^{-3}	6	10	6%	3×10^{-2}	4	10	171	4%
antigen processing and presentation of exogenous peptide antigen	4×10^{-3}	6	10	6%	2×10^{-2}	5	10	164	4%
antigen processing and presentation of exogenous peptide antigen via MHC class I, TAP-independent	6×10^{-7}	101	6	4%	2×10^{-2}	38	4	8	2%
antigen processing and presentation of peptide antigen	8×10^{-3}	5	10	6%	4×10^{-2}	4	10	181	4%
cellular response to interferon-gamma	2×10^{-3}	8	9	5%	1×10^{-2}	7	8	82	3%
cellular response to type I interferon	9×10^{-5}	17	8	5%	4×10^{-2}	7	6	65	3%
chromosome organization involved in meiosis	2×10^{-3}	15	6	4%	1×10^{-2}	13	6	36	3%
genetic imprinting	6×10^{-3}	16	5	3%	1×10^{-2}	17	5	22	2%
interferon-gamma-mediated signaling pathway	1×10^{-3}	10	8	5%	1×10^{-2}	9	7	61	3%
meiosis	4×10^{-2}	5	9	5%	4×10^{-2}	5	9	147	4%
meiosis I	2×10^{-2}	8	7	4%	2×10^{-2}	7	7	76	3%
positive regulation of adaptive immune response	1×10^{-3}	10	8	5%	4×10^{-2}	7	6	61	3%

Table S5 (continuation). Biological processes enriched in differentially methylated regions between MT-Group2 and WT by the GREAT software.

Term name	Binom FDR Q-value	Binom Fold Enrichment	Binom Observed Region Hits	Binom Region Set Coverage	Hyper FDR Q-value	Hyper Fold Enrichment	Hyper Observed Gene Hits	Hyper Total Genes	Hyper Gene Set Coverage
positive regulation of adaptive immune response based on somatic recombination of immune receptors built from immunoglobulin superfamily domains	6×10^{-4}	11	8	5%	3×10^{-2}	8	6	58	3%
positive regulation of cell killing	2×10^{-3}	11	7	4%	4×10^{-2}	9	5	42	2%
positive regulation of leukocyte mediated cytotoxicity	2×10^{-3}	12	7	4%	3×10^{-2}	10	5	39	2%
positive regulation of leukocyte mediated immunity	1×10^{-3}	10	8	5%	4×10^{-2}	7	6	61	3%
positive regulation of lymphocyte mediated immunity	1×10^{-3}	11	8	5%	4×10^{-2}	8	6	59	3%
positive regulation of T cell mediated cytotoxicity	1×10^{-4}	25	7	4%	1×10^{-2}	15	5	26	2%
positive regulation of T cell mediated immunity	7×10^{-5}	18	8	5%	1×10^{-2}	12	6	39	3%
regulation of T cell mediated cytotoxicity	3×10^{-4}	18	7	4%	2×10^{-2}	12	5	31	2%

Figure S1. Quality control for Illumina Infinium Human Methylation 450k arrays. **(A)** Mean detection *p*-values for each sample. **(B)** Density 'bean' plots (beta-values) for each sample.

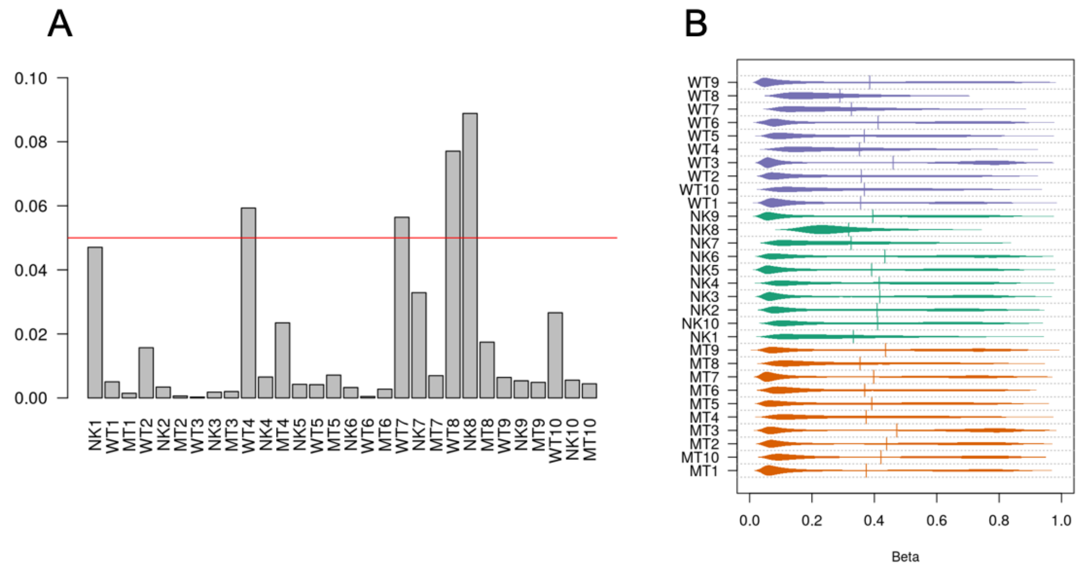

Figure S2. Density plots (beta-values) for raw data (left panel) and Quantile normalization procedure (right panel).

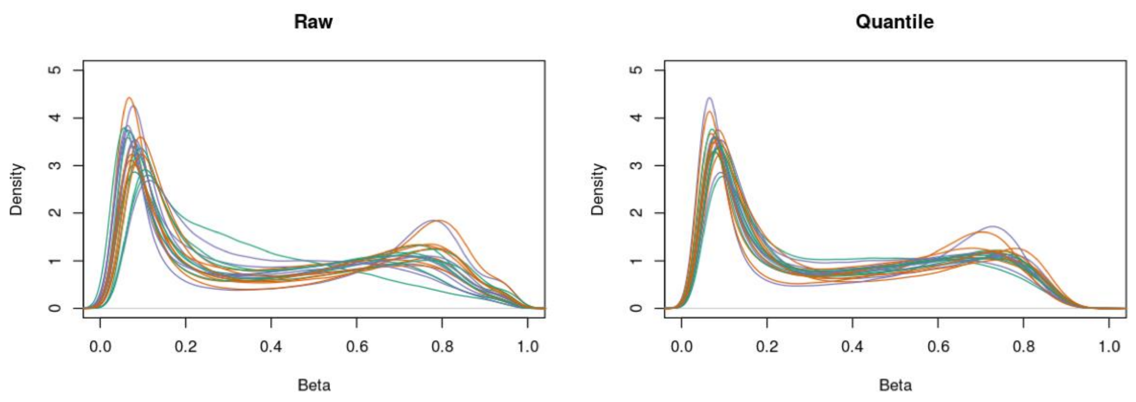

Figure S3. Boxplots of gene expression levels of NK, WT, MT-Group1 and MT-Group2 per gene with negative correlated between methylation and expression.

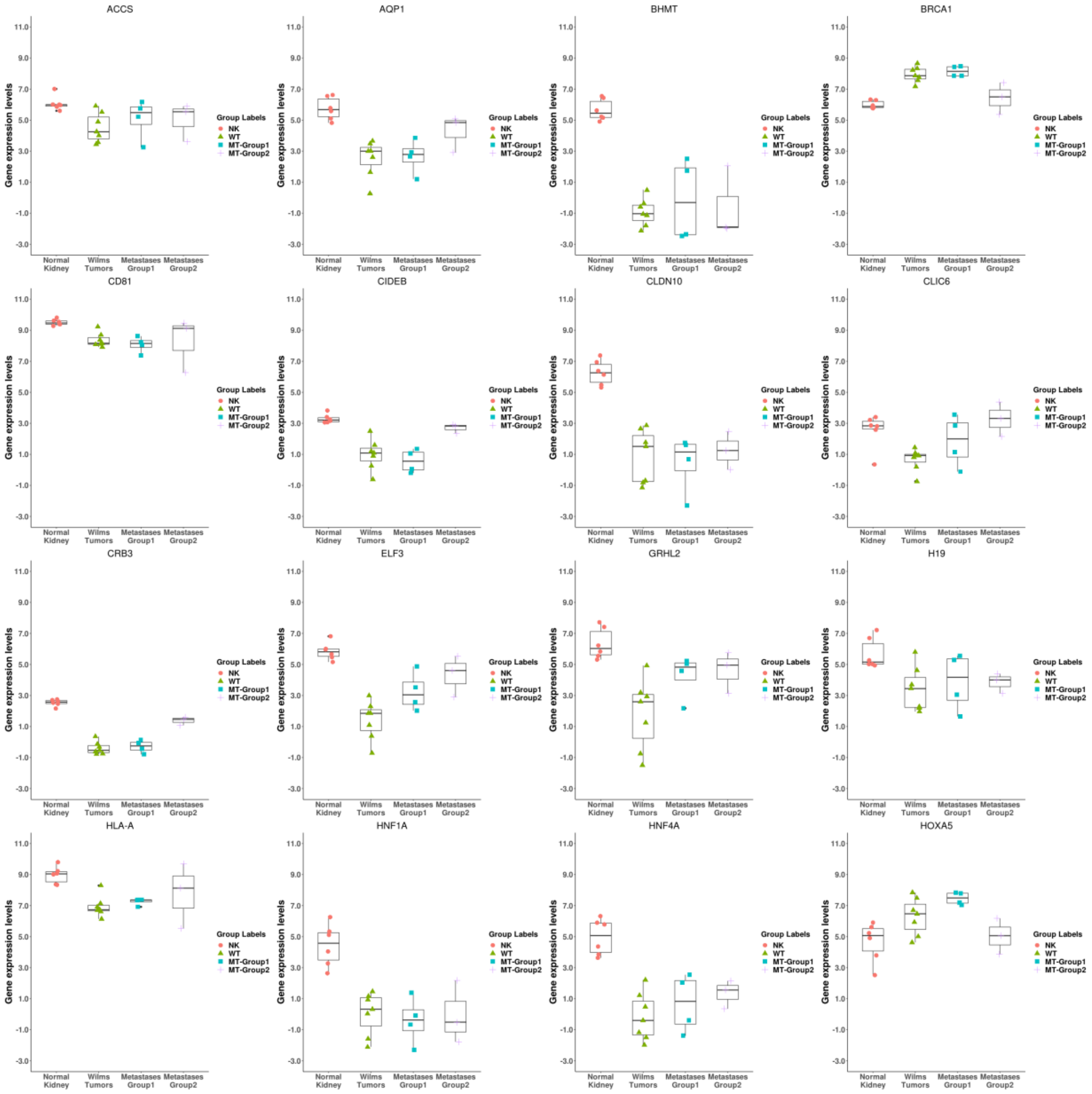

Figure S3 (continuation). Boxplots of gene expression levels of NK, WT, MT-Group1 and MT-Group2 per gene with negative correlated between methylation and expression.

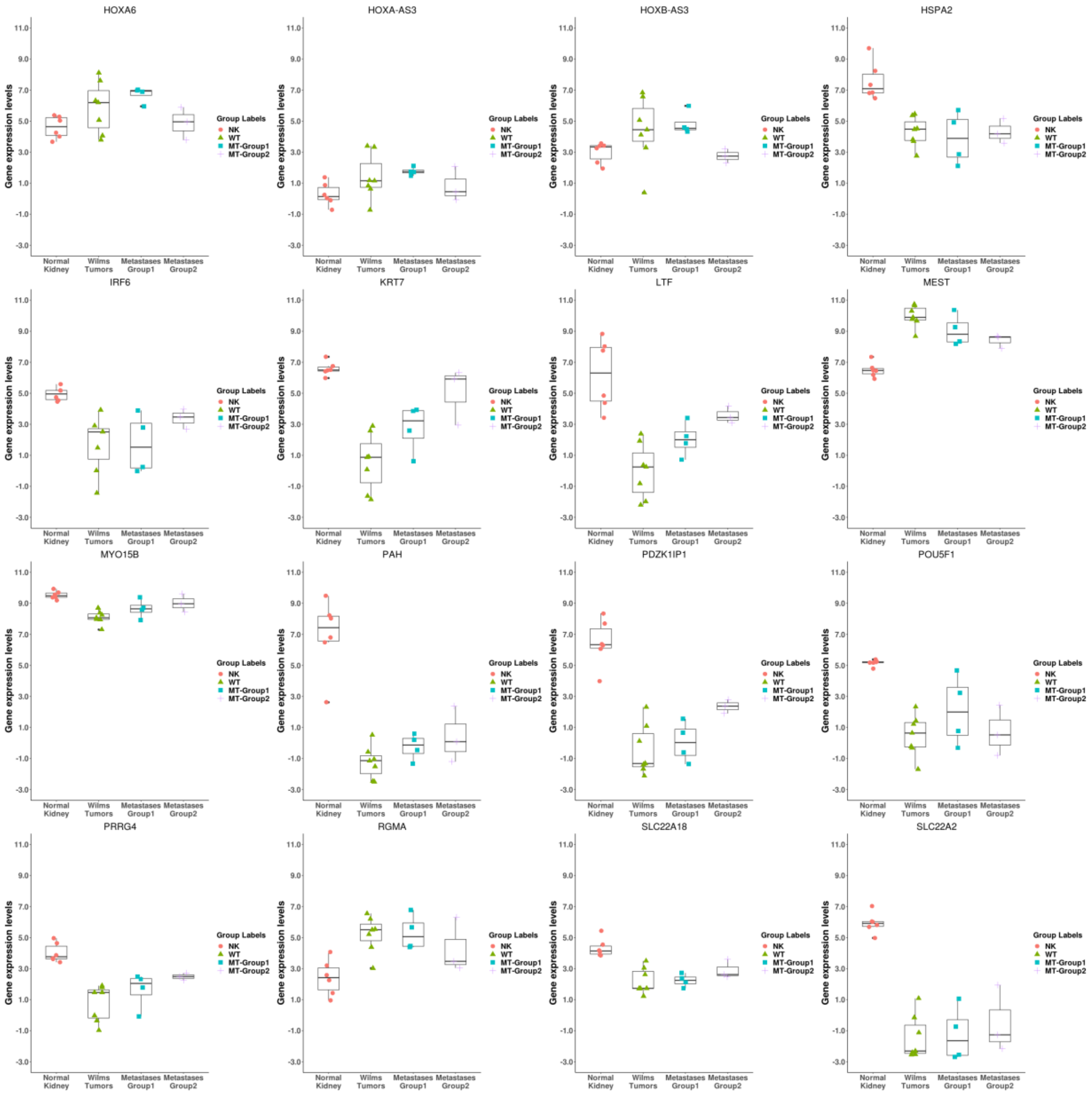

Figure S3 (continuation). Boxplots of gene expression levels of NK, WT, MT-Group1 and MT-Group2 per gene with negative correlated between methylation and expression.

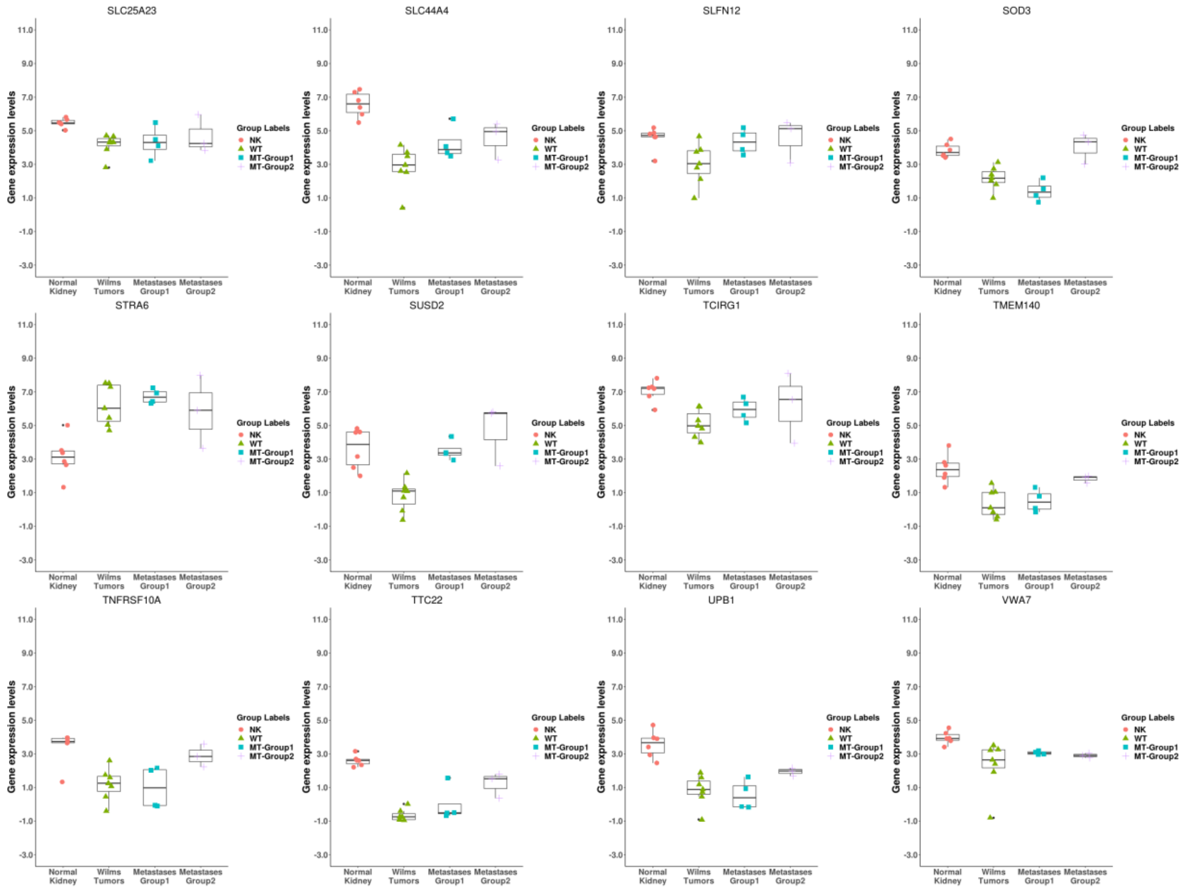

Supplement: Supplementary file 1 [file cells-08-00921-s001.pdf]
